# Complex routes into HIV care for migrant workers: a qualitative study from north India

**DOI:** 10.1080/09540121.2015.1114988

**Published:** 2015-11-26

**Authors:** Tanvi Rai, Helen S. Lambert, Helen Ward

**Affiliations:** ^a^ School of Public Health, Imperial College London, London, UK; ^b^ School of Social and Community Medicine, University of Bristol, Bristol, UK

**Keywords:** Transients and migrants, HIV infections/prevention & control, India, qualitative research, care pathways

## Abstract

Migrant workers are designated a bridge population in the spread of HIV and therefore if infected, should be diagnosed and treated early. This study examined pathways to HIV diagnosis and access to care for rural-to-urban circular migrant workers and partners of migrants in northern India, identifying structural, social and individual level factors that shaped their journeys into care. We conducted a qualitative study using in-depth interviews with HIV-positive men (*n* = 20) and women (*n* = 13) with a history of circular migration, recruited from an antiretroviral therapy centre in one district of Uttar Pradesh, north India. Migrants and partners of migrants faced a complex series of obstacles to accessing HIV testing and care. Employment insecurity, lack of entitlement to sick pay or subsidised healthcare at destination and the household's economic reliance on their migration-based livelihood led many men to continue working until they became incapacitated by HIV-related morbidity. During periods of deteriorating health they often exhausted their savings on private treatments focused on symptom management, and sought HIV testing and treatment at a public hospital only following a medical or financial emergency. Wives of migrants had generally been diagnosed following their husbands' diagnosis or death, with access to testing and treatment mediated via family members. For some, a delay in disclosure of husband's HIV status led to delays in their own testing. Diagnosing and treating HIV infection early is important in slowing down the spread of the epidemic and targeting those at greatest risk should be a priority. However, despite targeted campaigns, circumstances associated with migration may prevent migrant workers and their partners from accessing testing and treatment until they become sick. The insecurity of migrant work, the dominance of private healthcare and gender differences in health-seeking behaviour delay early diagnosis and treatment initiation.

## Introduction

Recent advances in biomedical prevention suggest that early initiation of antiretroviral therapy (ART) could stall the HIV epidemic; the first step is to reduce the proportion of people who are undiagnosed (World Health Organisation, 2013). In India, despite universal free testing and treatment, only 10–20% of people living with HIV are diagnosed (Steinbrook, 2007); together with poor linkage into care, this results in late presentation at ART centres (Sarna, Bachani, Sebastian, Sogarwal, & Battala, 2010).

Migrants have been considered at risk of acquiring HIV infection due to the social disruption of migration (Decosas, Kane, Anarfi, Sodji, & Wagner, 1995) and as bridging populations linking asynchronous epidemics (Coffee, Lurie, & Garnett, 2007). Migration and mobility are shaping many of the Asian epidemics (UNAIDS, 2008), for example, in western Nepal, associated with migration to Mumbai (Nepal, 2007), and in China, associated with rural-to-urban labour migration (Zhang, Chow, Jahn, Kraemer, & Wilson, 2013).

In India, rural-to-urban migration is rising (Abbas & Varma, 2014), with conservative estimates of around 40 million involved in circular migration (Srivastava, 2011). In north India, long-distance circular out-migration of men is widespread; if married, the man's wife usually remains at her marital home to look after children and elders. At destination men live in shared rooms and visit their villages once or twice a year (Srivastava, 2005). HIV “hot spots” are appearing within previously low-prevalence states which also report high out-migration, and are believed to result from this circulation of migrant workers between low-prevalence rural and high-prevalence urban areas, leading to nationwide awareness programmes targeting migrant families (NACO, 2010, 2014a).

As part of a larger mixed-methods study of HIV and migration in northern India (Rai et al., 2014; Rai, Lambert, & Ward, in press), we use a social ecological approach (Poundstone, Strathdee, & Celentano, 2004; Sweat & Denison, 1995) to report results from the qualitative study identifying how a multi-level set of factors combine in shaping journeys into care for migrant families with HIV.

## Methods

With 200 million people, Uttar Pradesh is the most populous state in the country with the highest levels of net out-migration (Census, 2012). It is a low-prevalence state but includes five high-prevalence districts^1^ (NACO, 2014b) including our study district, Allahabad.

Fieldwork was carried at the district-level ART centre in Allahabad over a period of six months in 2010–2011. The first author conducted face-to-face interviews in Hindi with 33 HIV-positive patients with a history of circular labour migration themselves or via their spouse. Study participants were sampled to achieve maximum variation in terms of age, gender, duration since HIV diagnosis and whether or not they were on ART. The topic guide included open-ended questions about their experience of migration, pathways to HIV diagnosis and life with HIV.

Interviews were translated and transcribed into English, managed using NVIVO v9 and analysed using Framework (Ritchie & Lewis, 2003) and thematic content analysis (Green & Thorogood, 2009). Ethical clearance was obtained from the Imperial College Research Ethics Committee and the local Institutional Ethics Review Board in Allahabad. Informed consent was obtained from all interviewees.

## Results

Twenty men and 13 women aged 24–45 were interviewed, including two couples. The men reported past or on-going migrant work in factories, transportation, construction, running small stalls or similar work. In keeping with local migration patterns, the women were wives or widows of migrant men rather than migrant workers themselves. Four men and five women had been diagnosed in the last six months, and the majority (24) were on ART.

**Figure 1.**
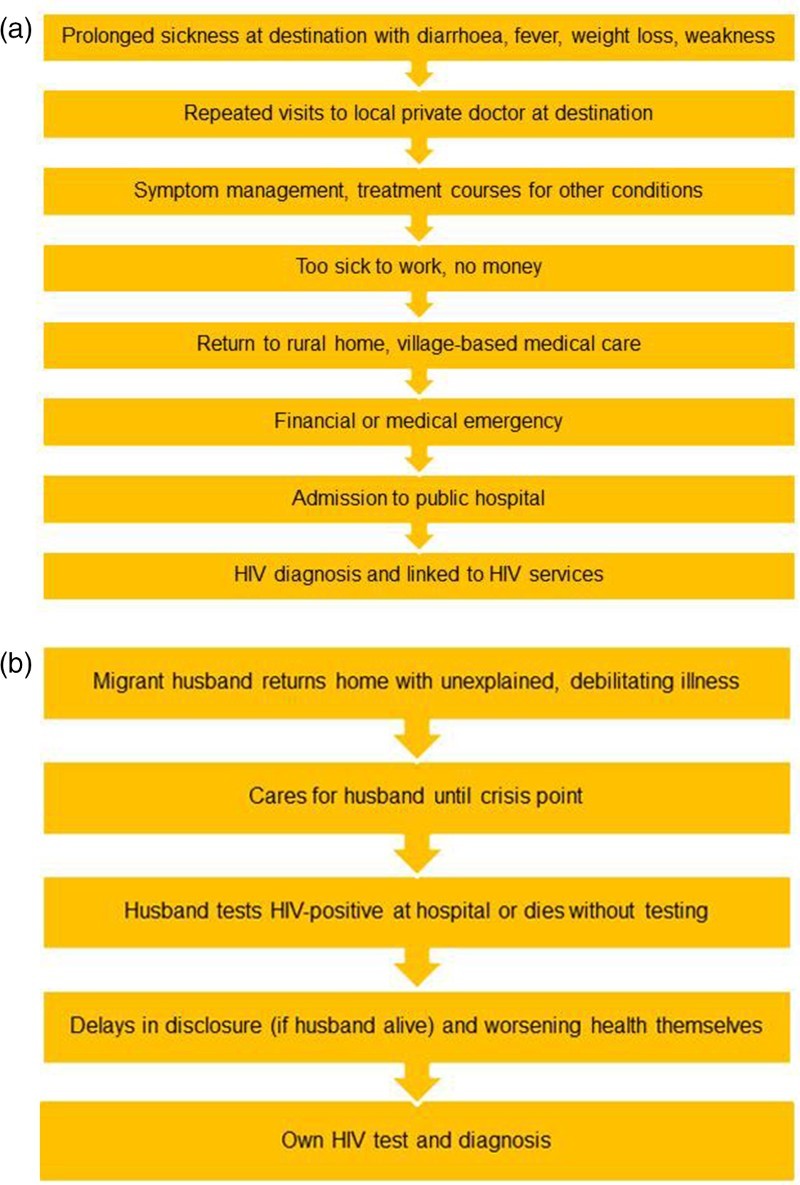
Routes to HIV diagnosis for migrant men and wives of migrant men. (a) Dominant pathway to testing for migrant men. (b) Dominant pathway to testing for wives of migrant men.

No participant had actively sought HIV testing; many had been aware of “AIDS” but without concern about personal risk. We identified three pathways to HIV testing and diagnosis presented in Table 1. The two dominant pathways, broadly coinciding with the experiences of male and female respondents, are illustrated in Figure 1(a) and 1(b) and detailed below.

**Table 1.** The three pathways to HIV testing and diagnosis.

•*In response to prolonged illness*, and no benefit from various treatments, usually obtained from private practitioners: largest group (*n* = 19), mostly men
•Following advice from relatives or friends *after a spouse was diagnosed HIV*-*positive*: second largest group (*n* = 11), mostly women
•Through *routine provider-initiated screening,* prior to a medical/surgical procedure (*n* = 2) or pre-emigration screening for when seeking work abroad (*n* = 1).

### Men's journeys of discovery

Men often worked 10–16-hour shifts, on informal contracts, paid by the hour or piece produced and generally not entitled to sick pay. Many talked of experiencing recurrent fever, diarrhoea and weight loss for months or years prior to their HIV diagnosis. They responded by visiting private practitioners who offered them symptomatic relief quickly at low cost.

“ … somebody who is a worker, he will try to get better for less money, he'll try to get better for that, 50 rupees. … So that's why … everybody goes there [cheap local practice] … ”. (ID 28)

Several respondents described being diagnosed and treated for several conditions.

“I had a fever constantly for a month, month and a half. Someone said I'd got typhoid, somebody else said I'd got malaria … ”. (ID 22)

While some private practitioners treated patients for other conditions, not testing for HIV until later on, others apparently tested for HIV but did not disclose it straightaway. Some told patients they had HIV but not about the free ART available at public hospitals, so patients continued to seek treatment at their own expense. It was unclear whether any of these treatments were ART.

“But then I became sicker … then some tests were done at a private clinic ( … ) it turned out to be HIV ( … ). But when those medicines were so expensive, I stopped taking them ( … ) Again I became very ill”. (ID 25)

Eventually, when migrant men were too debilitated to work, they returned home. Some continued seeking treatments from local doctors and healers. Only when their health collapsed or the money ran out completely, family members took them to public hospitals where they were diagnosed and referred to the ART centre.

“I was getting this fever, I'd just take some medicine ( … ) completely sickened, I moved back home. I got some treatment there in the local nursing home ( … ) When I was on my last legs they took me to [district hospital]. ( … ) it turned out to be HIV, and treatment started over there … ”. (ID 6)

Finally, more than half of the men's wives were HIV-positive, and two men's wives remained untested – these men, concerned about the social implications of having HIV, had chosen to remain quiet.

### Women's journeys of discovery

Women in this study had accessed the ART centre via their husbands or other male family members. The dominant family form in rural Uttar Pradesh is the patriarchal and patrilineal family, where women's lives are restricted to normatively defined “female” domains that lie within the household. Their exposure to information, and movement outside the home is strictly managed by male and/or older relatives (Jeffery & Jeffery, 1997). In this study, the low status of women in the marital household kept them unaware of their personal risk of HIV and they did not receive testing or treatment until facilitated by male relatives. Their migrant husbands being absent, or sick (or dying) from HIV-related illness contributed to the delay.

All but one^2^ of the women interviewed had been diagnosed following the death or diagnosis of their husbands. The husbands of widows had either failed to tell their wives about their HIV and had subsequently died, or in one case, the woman found out just before her husband's death.

“ … when he was in a bad state, then I was called to Bombay. So I went there ( … ) when I saw the report, he had it. … And then they got me tested too”. (ID 16)

Widows with HIV found themselves in a situation where there they had scant understanding of their condition and minimal emotional and financial support from their marital families. For many like the men, eventual contact with the ART centre was precipitated by a medical or financial emergency.

“My whole body was swollen, I went yellow, I couldn't walk, my body hurt ( … ) I first went to the government clinic ( … ) then I went to a private clinic. When I didn't get any relief they sent me here [ART centre]”. (ID 8)

Delayed disclosure was also reported by women whose husbands were alive. Migrating for work may have facilitated hiding their diagnosis, but even when men were sick at home other family members sometimes prevented their wives from accessing medical reports. A minority of women with living husbands had been more fortunate: their husbands' HIV discovery was followed quickly by their own HIV test when access to the ART centre had already been secured via their husbands.

## Discussion

Despite targeted migrant awareness campaigns and free HIV testing and treatment, people in this study were mostly unable to access these services until faced with some kind of medical or financial crisis intimately linked to their status as migrants or migrants’ wives. Delays in HIV testing postponed access to ART and prolonged the period during which they could transmit HIV to others in their sexual networks (Rai et al., 2014).

**Figure 2.**
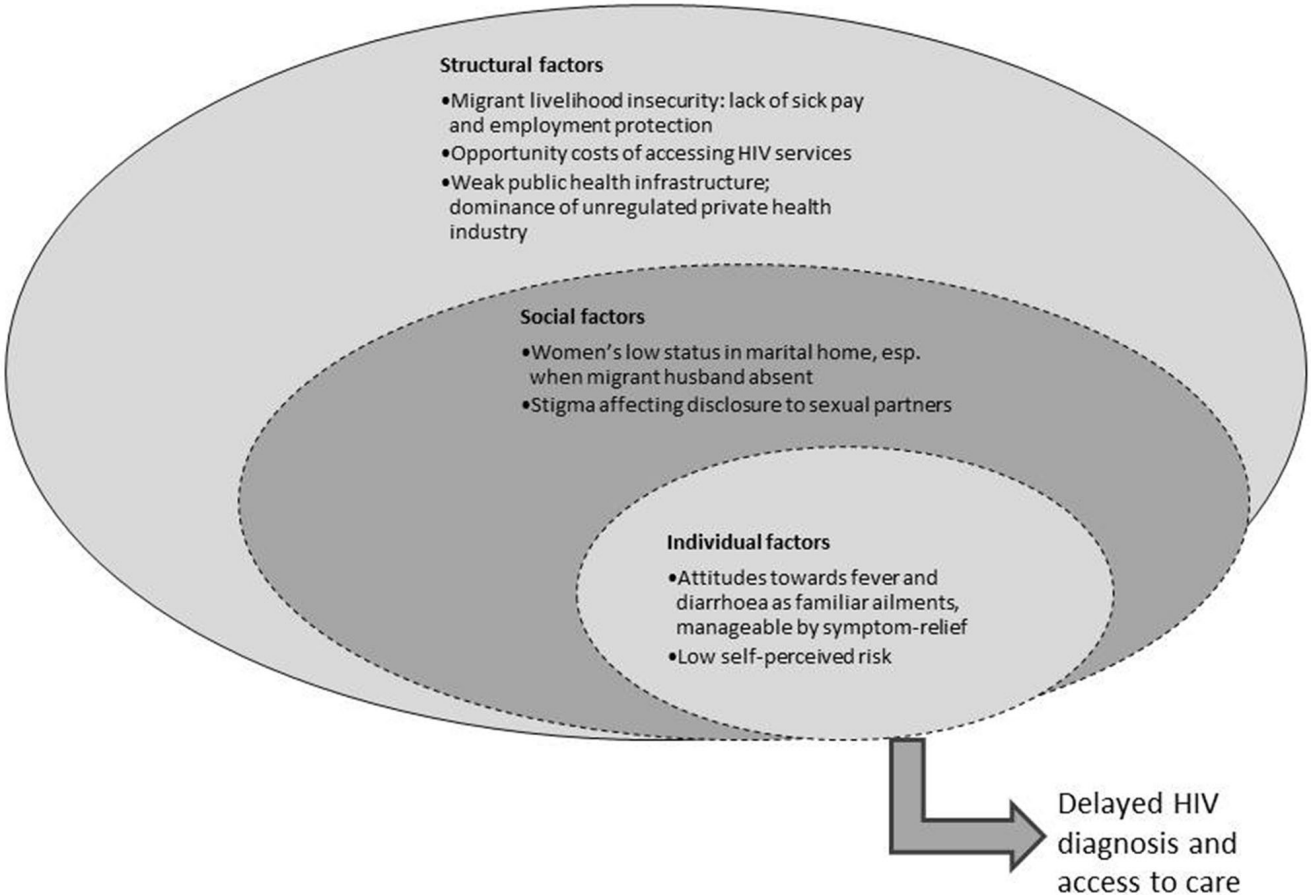
The structural, social and individual factors delaying HIV diagnosis and access to care for HIV-positive migrant workers and their partners.

Figure 2 represents our theoretical framework for understanding migrant families’ convoluted pathways into care.

*Structural factors*: The precarious nature of migrant work with its long shifts, informal employment contracts and lack of sick pay (Deshingkar & Akter, 2009; Faetanini & Tankha, 2013) created significant opportunity costs of seeking medical care leading men towards quick, inexpensive symptom-management solutions. The dominance of private health care is partly attributed to its reputation as more efficient, convenient and confidential than public sector services (Bhat, 1999; Sheikh, Porter, Kielmann, & Rangan, 2006). However, the medical mismanagement respondents faced whereby they were treated for many other conditions before HIV was diagnosed echoes previous research where private practitioners were frequently found not following current HIV guidelines (Chomat et al., 2009; Datye et al., 2006; Kielmann et al., 2005).

*Social factors*: This study supports previous research that marriage is the main risk factor for HIV (Gangakhedkar et al., 1997; Saggurti & Malviya, 2009), and that aside from antenatal HIV screening, married women are usually tested following their husband's HIV diagnosis (Joseph et al., 2010; Malave, Ramakrishna, Heylen, Bharat, & Ekstrand, 2013). Being a migrant's wife influenced both their risk of becoming HIV-infected and subsequent pathways into care. Women's low status in the marital home, made worse by absent husbands, kept them ignorant of their HIV risk and delayed access to care when unwell. Similar to other studies on partner notification (Chandra, Deepthivarma, & Manjula, 2003; Taraphdar, Dasgupta, & Saha, 2007), stigma about having a socially disgraceful disease such as HIV contributed to some men's hesitation in telling their wives.

*Individual-level factors*: Fever, diarrhoea and weight loss are the usual manifestations of conditions such as typhoid, malaria, tuberculosis and dengue – all highly prevalent illnesses in India, especially for migrant workers living and working in congested, unhygienic places such as urban slums and factories (Borhade, 2011; Chatterjee, 2006) and therefore study respondents not making the link to HIV is understandable. Seeking quick and accessible symptomatic relief for these familiar and frequently experienced health conditions, sometimes repeatedly, rather than going to a public hospital (where there is a greater likelihood of being referred to HIV services) may be especially applicable to circular migrant workers who are in their work locations for finite periods and are often not entitled to sick pay (Deshingkar & Akter, 2009).

Extensive awareness campaigns at source, transit and destination (NACO, 2010) may reduce the delays into care for HIV-positive migrant families but they largely neglect many of the structural and social factors that make HIV-positive migrant families particularly vulnerable. We could only sample those who were accessing ART services but the treacherous journeys of these ‘lucky’ individuals give some suggestion of the plight of those not yet on the ART register.

## Notes

1. Areas with an estimated antenatal HIV prevalence of >1%.

2. The remaining woman was diagnosed following illness after her marriage dissolved.
